# Evolution led humans to bipedalism, but we live in a sedentary society: Will “Sunday running” protect us from NCDs at no cost?

**DOI:** 10.3389/fpubh.2022.1031911

**Published:** 2023-01-06

**Authors:** Valentina Presta, Giuliana Gobbi, Giancarlo Condello, Cecilia Carubbi, Elena Masselli, Prisco Mirandola, Marco Vitale

**Affiliations:** ^1^Department of Medicine and Surgery, University of Parma, Parma, Italy; ^2^Curriculum of Sport Sciences & Human Health, University of Parma, Parma, Italy; ^3^Movement Analysis Laboratory (LAM), Parma University Hospital, Parma, Italy

**Keywords:** running, walking, aging, sedentary behavior, health promotion

## Abstract

Evolution led humans to bipedal stance and movement. However, we live in a sedentary society that strongly challenges our willingness to be physically active. We (mis)understand that being at least a Sunday runner could protect us from sedentary-related diseases, but what if this compromises the healthier life expectancy anyway? Citing Paul Gauguin, we know where we come from and what we are, the question arises about where we are going. And also, how.

…*We were born to run…* rocked out Bruce Springsteen in 1975, 47 years ago, while almost 20 years have passed since Bramble and Lieberman (1) reviewed the scientific literature to discuss the endurance running performance of bipedal gait in the light of the evolutionary theory. *Nature* covered the issue with an emblematic *Born to Run* linked with the hypothesis expressed by the authors: endurance running capabilities derive from the divergence between the genus *Homo* and quadrupeds lineages. Bipedalism is undoubtedly an essential chapter of the evolutionary history of humans (2). Given the absence of a satisfactory genetic basis for the quadrupedal-bipedal transition, all theories regarding bipedal locomotion are justified by the necessity to explore new habitats and new possibilities for hunting activities and food search (instrument handling while moving). Bipedalism thus became the opportunity to cover long distances, explore, and survive other species. The paradox of 2 million years of human evolutionary history tells us that we evolved by *choosing* to move, but now we *must* move.

Living amid the continuous development of new technologies and scientific discoveries, our routine is simplified, fast, and rapidly accessible. Our everyday life is easier and safer than before. Is it also healthier? Daily, we accumulate (unnatural) sedentary hours in contemporary activities (e.g., work-related activities, reading, watching TV, gaming, etc.) (3). Sedentary behavior is known to be a risk factor for many non-communicable diseases (NCDs), such as cardiovascular, metabolic, cancer, etc. By contrast, physical activity is part of prevention strategies against NCDs, together with healthy nutrition and lifestyles. Engaging in regular physical activity (by playing sports and being as active as possible during the daily routine) is proven to be a protective factor against NCDs and NCDs-related risk factors, namely overweight and obesity, and hypertension. Improvement in mental health, quality of life, and wellbeing are widely reported across all ages for people meeting the recommended levels of physical activity (e.g., in adulthood, it is recommended a weekly amount of 150–300 min of moderate-intensity and 75–150 min of vigorous-intensity physical activity in combination with muscle-strengthening activities) (4, 5). World Health Organization (WHO) Guidelines on physical activity and sedentary behavior (5) made recommendations to fight sedentary lifestyles according to different age ranges and clinical- and gender-related (i.e., pregnancy) conditions. Among these, everybody knows the daily goal of 10,000 steps. Although it is the minimum to become active, it emphasizes the message of accumulating physically active moments in a day contributing to the beneficial effects of staying active, like body weight management, improvement of physical fitness and mental health, and the reduction of NCDs-related risk factors.

So, we evolved to move, and now we must move. Running or walking? *That's the question*: are we runners or walkers by nature? (Figure 1).

**Figure 1:**
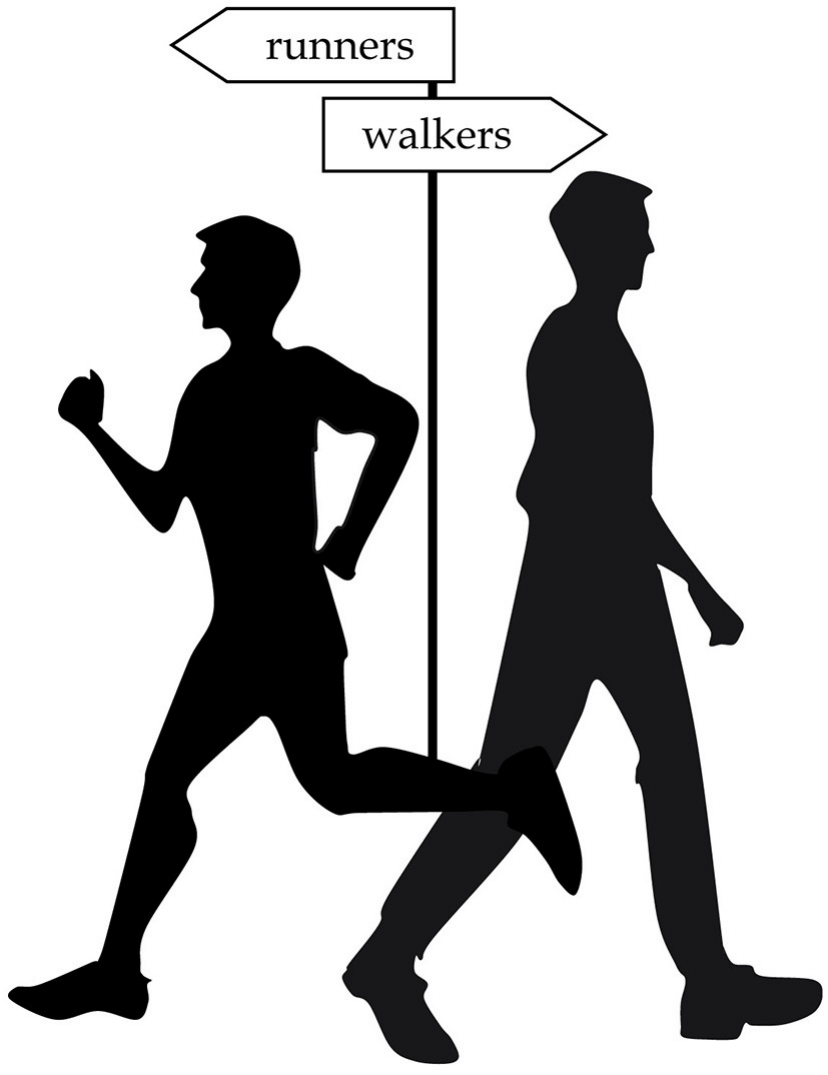
Are we runners or walkers by nature?

To go back to the thesis that we were *born to run*, let us consider an evolutionary perspective. Bipedalism allowed the rescue of the forelimb from weight support, and thus dexterity acquisition–which was worth the huge neurological costs of a bipedal stance (6). By studying fossils and morpho-functional adaptations of humans, we only know for sure that they were habitual walkers, even for long distances. Thus, we cannot discriminate if running was a stable behavior of the first hominids or a further evolution of walking capabilities. Running would be considered as an optimization of walking for a better life in the wild, aimed at covering long distances (by managing the running energy cost and improving both mechanical and thermoregulatory systems, such as joint stabilization and sweat dissipation, respectively). Further, the main activities of the early hominids, such as hunting, predator survival, and food search, forced humans to evolve their gait technique to go faster and farther—thus, running. So, does the evolutionary hypothesis sustain a “running path” rather than a “walking path”?

*Run forrest run*: running grew quickly around the world in the last decades, both as a professional athletic sport and as a hobby or form of exercise, with novices or non-professionals opting for this activity because it is a faster and more accessible way to reduce sedentary behavior in one's daily routine. However, running jeopardizes its own future by increasing the risks of overuse damage and traumatic injuries (7). Injury risks notably increase with previous injury history and long distances (marathons and half-marathons) covered. Aging by itself increases the injury risk in short-distance runners (8). Joint overload/overuse, especially in the 45–65 age range, is associated with the risk of injuries and age-related comorbidities, hampering the life-long overall amount of physical activity and paradoxically increasing inactivity relapse (9–11). Therefore, if we consider running-related injuries from the perspective of a longer life expectation (damage accumulation), should we not indeed preserve our joints and avoid overuse and trauma? If running jeopardizes our future as runners, will walking preserve our future as walkers?

Should we run or walk? Is running a *panacea* for a sedentary lifestyle? The life expectancy of our ancestors was not more than 40 years (12); running brought more advantages than damages and our anatomy evolved allowing efficient running (13). Now that we live twice as long, walking rather than running seems like the opportunity to grow and go farther. *Alone a youth runs fast, with an elder slow, but together they go far*. Unless you must escape from a predator. If so, run.

## Data availability statement

The original contributions presented in the study are included in the article/supplementary material, further inquiries can be directed to the corresponding author.

## Author contributions

MV conceived the study. VP and GG drafted the manuscript. GC, CC, EM, and PM edited the final manuscript. All authors contributed to the article and approved the submitted version.
